# The General Psychopathology Factor: Structural Stability and Generalizability to Within-Individual Changes

**DOI:** 10.3389/fpsyt.2019.00594

**Published:** 2019-08-30

**Authors:** Kia Gluschkoff, Markus Jokela, Tom Rosenström

**Affiliations:** ^1^Department of Psychology and Logopedics, Faculty of Medicine, University of Helsinki, Helsinki, Finland; ^2^Department of Mental Disorders, Norwegian Institute of Public Health, Oslo, Norway

**Keywords:** general psychopathology factor, p factor, comorbidity, bifactor model, invariance

## Abstract

**Objectives:** Although cross-sectional investigations have found a bifactor structure of psychiatric comorbidity that includes a general psychopathology factor plus more specific factors, prospective evidence supporting the bifactor structure is still limited. We evaluated the structural stability (i.e., longitudinal invariance) of the bifactor model in comparison to an alternative structure, a correlated factors model without a general psychopathology factor. We also investigated the models’ generalizability to change processes in psychopathology.

**Methods:** The analyses were conducted on 10-year follow-up data from 5,001 respondents in the US National Comorbidity Survey. Invariance was evaluated through a series of nested invariance tests using confirmatory factor analysis, and the models’ generalizability to change processes was investigated using change scores of disorder status.

**Results:** The bifactor model and the correlated factors model exhibited an equal degree of strong structural stability over time. Only the bifactor model satisfactorily characterized the structure of temporal changes in psychopathology.

**Conclusions:** The bifactor structure with a general psychopathology factor is stable over time and describes temporal changes in psychopathology. The findings support the notion that the general psychopathology factor describes a transdiagnostic etiology and may therefore provide a useful target for intervention and treatment.

## Introduction

Extensive comorbidity of psychiatric disorders has been well established by epidemiological studies. Early psychometric models suggested that such comorbidity would be accounted for by latent transdiagnostic factors of internalizing (mood and anxiety disorders) and externalizing (substance use, impulsivity, and antisocial behavior) but could not explain why externalizing and internalizing traits were also “comorbid” (i.e., co-occurring). More recent research has explored a bifactor model of psychopathology that includes a latent general psychopathology factor, sometimes labeled the *p factor* (1). The bifactor model thus consists of a broad general psychopathology factor that is presumed to underlie all psychiatric disorders and conceptually narrower specific factors of internalizing and externalizing psychopathology. The general psychopathology factor captures what is common across all forms of psychiatric diagnoses and accounts for the co-occurrence of internalizing and externalizing disorders.

Even though the bifactor model has been empirically supported across a range of diverse samples (1–7), significant debate remains regarding the metastructure of psychopathology and the validity of the general psychopathology factor. A central issue is whether the overall structure of psychopathology should be modeled as a) a bifactor model with a broad general psychopathology factor and narrower specific factors that are not correlated or b) with a model that includes multiple correlated factors but no general factor of psychopathology. The externalizing and internalizing traits could co-occur due to mutual interaction, not due to a latent general factor. However, finding that a general factor model fits the data does not prove the factor’s substantive existence because a general factor could emerge simply because data features positive correlations among disorders (8). Furthermore, researchers have raised concerns regarding the bifactor model’s tendency to show superior performance in terms of model fit, suggesting that the bifactor model may be inclined to capture random noise in the data (9, 10).

Overall, despite the surge of interest in the general psychopathology factor and recent advances in understanding the structure of psychopathology, many pertinent questions regarding the nature of the bifactor model remain unanswered. Most studies examining the bifactor model have measured psychopathology at a single point in time, thereby providing an incomplete picture on the model’s longitudinal properties and on the development of psychiatric comorbidity. Although a few studies have investigated the temporal stability of the bifactor model (11–13), the model’s longitudinal invariance, that is, structural stability, has not been formally tested. Whereas temporal stability describes the extent to which the model’s latent factors predict each other at a later time point, longitudinal invariance denotes that the model’s latent factors have the same meaning over time. Establishing longitudinal invariance is thus a prerequisite to making meaningful comparisons of factor means over time. It is currently not known whether the bifactor model parameters such as factor loadings are temporally consistent or, in other words, if the relationships between psychiatric disorders and the latent factors of the bifactor model remain the same across successive measurement occasions of same individuals. Because the factors are indirectly observed latent entities, it is important to ensure they exist in a comparable form with the same meaning over time. Another question to be explored is whether the bifactor model is structurally more or less stable than for example, the traditional correlated factors model. Testing the longitudinal invariance of the bifactor model and comparing it with the invariance of a competing model without a general psychopathology factor may provide important insights into underlying etiology and into the models’ applicability.

Although longitudinal invariance establishes an age-invariant factorial structure for *between*-individual differences in the population, allowing one to interpret change in the factors, it gives no direct guarantees that *within*-individual changes follow the same structure (14). Discovering that the same longitudinally invariant structure generalizes to data on temporal changes would add evidence for a hypothesis that the modeled latent factors are situated within individuals, driving parallel changes in multiple disorders. If disorders across internalizing and externalizing spectrums change in the same direction within individuals (i.e., individuals experience parallel onsets or recoveries from disorders from both spectrums), this suggests that a general factor is driving the process by which psychiatric disorders develop across time. In contrast, if changes in internalizing disorders are not typically accompanied by changes in externalizing disorders (beyond chance rate), then within-individual change in psychopathology is not driven by a general factor but rather by underlying internalizing and externalizing factors or by disorder-specific influences. Furthermore, even if disorders across spectra do exhibit parallel changes over time, it becomes appropriate to ask whether this “parallelism” is better described by a bifactor structure or, for example, by correlated internalizing and externalizing factors. According to previous work, internalizing and externalizing factors explain the comorbidity in the onset of psychiatric disorders (15). However, whether the general psychopathology factor better accounts for such comorbidity remains to be investigated.

The central unanswered question here is whether individual differences in intraindividual patterns of change in psychiatric comorbidity can be characterized by a similar factor structure as pointwise cross-sectional individual differences. Assuming that patterns of potential confounding factors for cross-sectional individual differences do not carry over to change processes, such a finding would suggest that the latent factors reflect true underlying psychopathology mechanisms. In other words, the longitudinal applicability of the general-factor model would suggest the existence of a transdiagnostic etiological factor that might respond to treatment. Successful treatment of such etiological factor would not only treat all psychiatric disorders instead of just one but would also allow us to understand cases where diagnostic “migration” is seen instead of recovery.

To sum, despite recent progress in understanding psychiatric comorbidity, much remains to be learned about the structure of psychopathology and the validity of the general psychopathology factor. Little is known about the structural stability of the bifactor model or how the model compares with other comorbidity models in prospective data. It is also currently unknown whether change in psychopathology follows a distinct structure. Compared to cross-sectional investigations, prospective studies have the potential to offer more convincing evidence on the structure of psychiatric comorbidity and are essential to enhance our knowledge about whether the general psychopathology factor is a substantive construct or merely a statistical artifact. Within this context, the primary purpose of the present study was to examine the longitudinal invariance of the bifactor model in comparison to a traditional correlated factors model and to evaluate the models’ generalizability to change processes in psychopathology, which has not been done before, as far as we know.

## Materials and Methods

### Sample

Data came from the 5,001 respondents who participated in the 1990–1992 National Comorbidity Survey (NCS) (16) and the 2001–2003 NCS follow-up survey (NCS-2). The baseline NCS was a nationally representative survey of US households designed to study the prevalence and correlates of Diagnostic and Statistical Manual of Mental Disorders (DSM)-III-R mental and substance disorders. The survey was administered to 8,098 respondents in the noninstitutionalized civilian population, with a response rate of 82.4%. The survey was administered in two parts. Part I included the core diagnostic interview and was administered to all respondents. Part II assessed additional disorders and was administered to a probability subsample of 5,877 including all respondents aged 15–24 years, all others with any lifetime DSM-III-R disorder assessed in Part I, and a random subsample of remaining Part I respondents. Further details about the NCS design are reported elsewhere (16).

The NCS-2 attempted to trace and reinterview all of the original Part II respondents a decade after the baseline NCS using an expanded version of the baseline interview that assessed onset, course, and severity of mental disorders between the two surveys. A total of 5,463 respondents were successfully traced, of whom 166 were deceased and 5,001 reinterviewed, yielding a conditional response rate of 87.6%. The unconditional response rate, i.e., the percentage of baseline respondents interviewed in both surveys, was 72.2% (0.876 × 0.824). Relative to the baseline, NCS-2 respondents were significantly more likely to be female, well-educated, and residents of rural areas. A nonresponse adjustment (a sampling weight) was used to correct for these discrepancies.

### Diagnostic Assessment

The baseline NCS assessed lifetime DSM-III-R disorders using a modified version of the World Health Organization Composite International Diagnostic Interview (CIDI), a structured interview administered by trained nonclinician interviewers (17). NCS-2 assessed psychiatric disorders present during the follow-up using a more recent version of the CIDI (18), which was based on the DSM-IV criteria. The interviews yielded psychiatric diagnoses and information about the course of disorders, such as age at first occurrence of symptoms.

The diagnoses considered were mania (MAN), generalized anxiety disorder (GAD), major depressive episode (MDE), dysthymia (DYS), posttraumatic stress disorder (PSTD), agoraphobia (AGO), panic disorder (PD), social phobia (SOP), specific phobia (SP), alcohol abuse (ALC), drug abuse (DRG), and antisocial personality disorder (APD). The diagnoses were operationalized without hierarchy rules and assessed with a 9-year recency criterion with the exception of APD, for which only lifetime diagnosis was available. The recency criterion was chosen to optimize coverage while avoiding temporal overlap between the two follow-ups. Bipolar disorder and conduct disorder were excluded from analyses because their recency could not be assessed at baseline and because their more severe variants (MAN and APD) were already included.

### Statistical Analyses

#### Cross-Sectional Factor Structure

As a preliminary analysis, exploratory factor analysis (EFA) was conducted to examine the cross-sectional factor structure of psychopathology at baseline (T1) and follow-up (T2) and to determine if the factor loadings were as expected based on previous literature. To establish the optimal number of factors to be extracted in EFA, parallel analysis was performed (19). After conducting EFA, confirmatory factor analysis (CFA) was conducted to investigate the fit of the possible factor structures. For completeness, we fitted and compared five different CFA models: 1) a one-factor model, 2) a two-factor correlated factors model with internalizing and externalizing factors, 3) a three-factor correlated factors model with fear, distress, and externalizing factors, 4) a bifactor model with a general factor and internalizing- and externalizing-specific factors, and 5) a bifactor model with a general factor and fear-, distress-, and externalizing-specific factors. Model fit for competing CFA models was assessed using the comparative fit index (CFI), Tucker–Lewis index (TLI), and root mean square error of approximation (RMSEA). Current conventions suggest that good fit is indicated by a CFI and TLI >0.95 and an RMSEA <0.06 (20), whereas adequate fit is indicated by a CFI and TLI >0.90 and an RMSEA between 0.06 and 0.08. We additionally report chi-square test values and degrees of freedom but omit *p* values because the test is oversensitive in large samples (21). Construct reliability was assessed with Hancock’s *H* (22), an index of latent construct reliability. All CFA models were estimated using Mplus version 7 (23) with mean- and variance-adjusted weighted least squares (WLSMV) estimation. For the best-fitting models, we also provide Akaike information criterion (AIC) and Bayesian information criterion (BIC) values obtained using robust maximum likelihood estimation (MLR) in the supplementary material.

#### Longitudinal Invariance

Longitudinal invariance was tested for the bifactor model and the correlated factors model. Instead of estimating separate models for each measurement occasion as in cross-sectional CFA, data from both occasions were combined for the invariance analysis and longitudinal CFA models with lagged correlations were fitted. For both the models tested, we first evaluated whether the same number of factors and the same pattern of factor loadings characterize the structure of psychopathology at T1 and T2 (configural invariance, i.e., the unconstrained model). The stability of factor loadings (weak invariance) was then examined by constraining all corresponding factor loadings to be equal across the two time points. Finally, threshold invariance (strong invariance) was tested by additionally constraining indicator thresholds across T1 and T2. With binary indicators, such as disorder statuses, the thresholds indicate the cut-point on an assumed unobserved continuum that provides the observed disorder prevalence. Delta parameterization was used in the model specification, and for purposes of model identification, factor means were constrained to zero and factor variances were fixed at unity. Lagged factor correlations, lagged indicator correlations, and measurement error variances (uniquenesses) were freely estimated.

When the sample size is large, the chi-square difference test is likely to detect trivial decrements in model fit as statistically significant (21, 24, 25). Consequently, we did not perform chi-square difference test but rather used the change in CFI (ΔCFI) as an empirically supported (24) criterion to compare models with different levels of invariance. Although there is no consensus on the standards by which significant decreases in model fit should be evaluated when testing for invariance with categorical indicators, a ΔCFI >0.01 has previously been suggested to indicate that invariance should be rejected (21).

#### Factor Structure of Change in Psychopathology

The structure of change in psychopathology was examined using change scores of disorder status. The scores were calculated for each individual disorder by subtracting the T1 disorder status (1 = yes, 0 = no) from the T2 disorder status. A score of −1 reflected disorder recovery, a score of 0 indicated no change in disorder status, and a score of 1 reflected disorder onset between T1 and T2. Again, correlations among underlying liabilities (risks) of these ordinal-valued changes were modeled rather than raw correlations.

## Results

### Cross-Sectional Factor Structure

**Figure 1:**
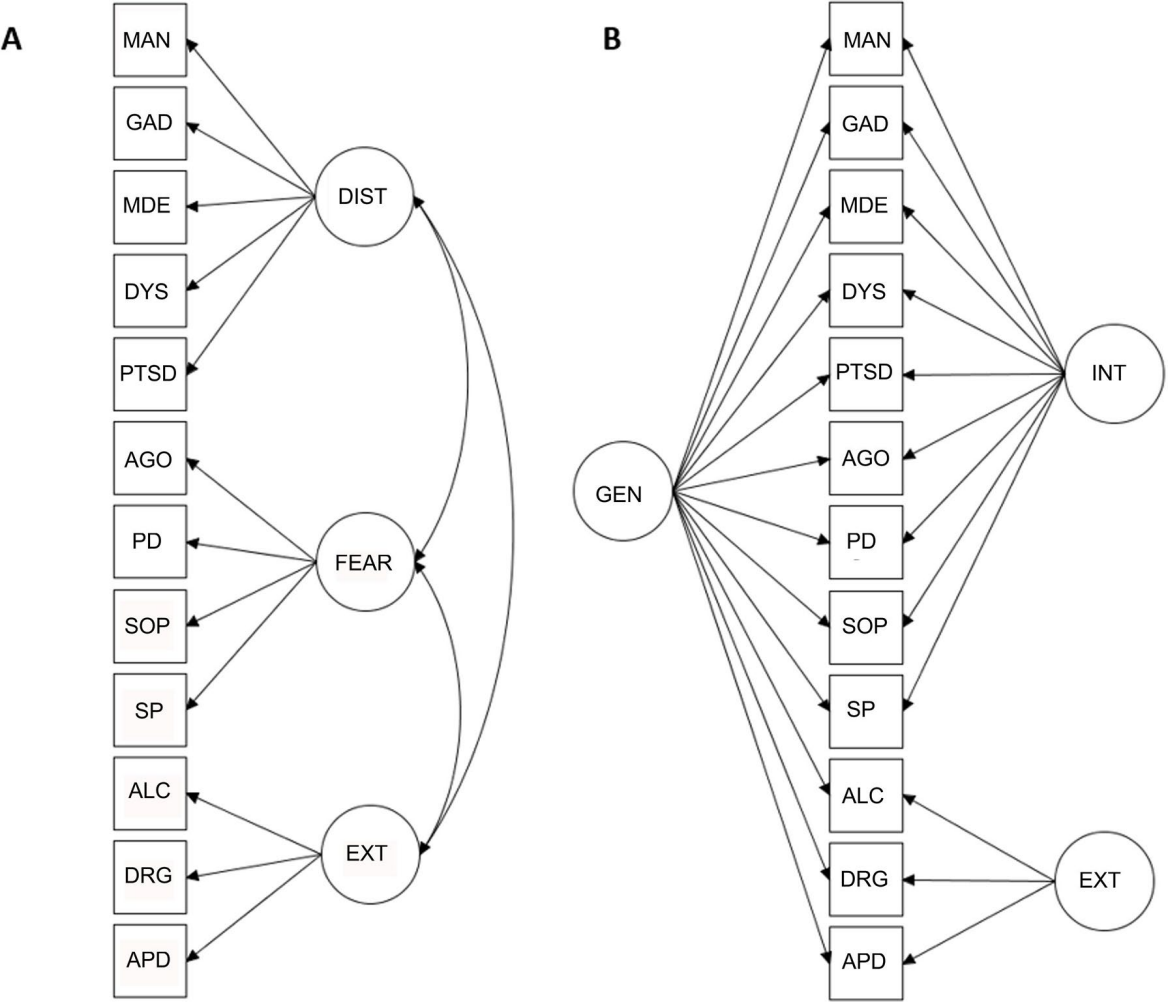
Simplified representations of the correlated factors model **(A)** and the bifactor model **(B)**. MAN, mania; GAD, generalized anxiety disorder; MDE, major depressive episode; DYS, dysthymia; PSTD, posttraumatic stress disorder; AGO, agoraphobia; PD, panic disorder; SOP, social phobia; SP, specific phobia; ALC, alcohol abuse; DRG, drug abuse; APD, antisocial personality disorder; DIST, distress; EXT, externalizing; INT, internalizing; GEN, general.

The detailed results from the preliminary cross-sectional analyses, including factor loadings from EFA and CFA, model fit indices for all CFA models tested, and construct reliability coefficients are presented in **Supplementary Tables 1**–**4**. Parallel analysis suggested three underlying factors for both T1 and T2 data. In confirmatory analyses, the bifactor model with a general factor and two specific factors showed superior fit over all other factor structures tested (CFI = 0.989, TLI = 0.983, RMSEA = 0.017 for T1). The second-best fitting model, a correlated three-factor model, also demonstrated good model fit (CFI = 0.978, TLI = 0.972, RMSEA = 0.022 for T1). In both the models, the latent factors showed good construct reliability, with the exception of the specific internalizing factor, which was not well defined, as it included both positive and negative loadings and some of the loadings were low in magnitude. Increasing the number of specific factors in the bifactor model (i.e., specifying separate factors for fear and distress) resulted in an inadmissible solution, potentially due to the extraction of too many factors. The bifactor model with two, rather than three, specific factors was thus selected for further analysis. A simplified representation of the two best fitting models is presented in **Figure 1**.

### Longitudinal Invariance

Given that the correlated three-factor model and the bifactor model had the best fit to cross-sectional data, we examined longitudinal invariance for these two factor models (**Table 1**). In the unconstrained longitudinal models with all parameters free to vary, both models had the same pattern of factor loadings over time (as indicated by good model fit at steps 1a and 1b of **Table 1**). In contrast to the longitudinal correlated factors model with positive loadings on each factor, the specific internalizing factor in the longitudinal bifactor model included both positive and negative loadings that represented mainly high distress and low fear (see **Table 2** for the unconstrained factor loadings). In addition to the invariant pattern of loadings over time, the magnitude of loadings was also invariant in both the correlated factors and the bifactor model (as indicated by the ΔCFI value of <0.01 at steps 2a and 2b).

**Table 1:** Longitudinal invariance of the correlated factors model and the bifactor model.

Model	Level of invariance	Chi-square (df)	RMSEA	TLI	CFI	ΔCFI
Correlated factors model	Step 1a (configural invariance)	547.728 (225)	0.017	0.965	0.971	
	Step 2a (loading invariance)	575.952 (237)	0.017	0.965	0.970	−0.001^a^
	Step 3a (threshold invariance)	683.391 (249)	0.019	0.957	0.961	−0.009^b^
Bifactor model	Step 1b (configural invariance)	366.433 (213)	0.012	0.982	0.986	
	Step 2b (loading invariance)	433.042 (237)	0.013	0.980	0.983	−0.003^a^
	Step 3b (threshold invariance)	534.756 (249)	0.015	0.972	0.975	−0.008^b^

^a^Step 2 compared to step 1, ^b^step 3 compared to step 2. Each disorder had only a single threshold that was a direct reflection of its prevalence rate, and, correspondingly, the time-specific thresholds were the same between the bifactor model and the correlated factors model.

**Table 2:** Unconstrained factor loadings (standard errors in parentheses) for the longitudinal correlated factors model and the longitudinal bifactor model.

Time	Disorder	Correlated factors model			Bifactor model		
		Distress	Fear	Externalizing	General	Internalizing	Externalizing
T1	MAN	0.804 (0.033)			0.767 (0.034)	*0.141 (0.090)*	
	GAD	0.745 (0.030)			0.707 (0.032)	0.179 (0.073)	
	MDE	0.833 (0.021)			0.785 (0.032)	0.345 (0.076)	
	DYS	0.729 (0.027)			0.674 (0.043)	0.430 (0.072)	
	PTSD	0.599 (0.035)			0.578 (0.034)	*0.024 (0.067)*	
	AGO		0.736 (0.033)		0.673 (0.042)	−0.359 (0.069)	
	PD		0.778 (0.034)		0.708 (0.032)	*−0.107 (0.080)*	
	SOP		0.704 (0.026)		0.644 (0.035)	−0.335 (0.065)	
	SP		0.749 (0.028)		0.682 (0.034)	−0.328 (0.066)	
	ALC			0.798 (0.026)	0.275 (0.030)		0.786 (0.030)
	DRG			0.922 (0.028)	0.346 (0.033)		0.846 (0.034)
	APD			0.744 (0.035)	0.400 (0.043)		0.567 (0.038)
T2	MAN	0.698 (0.039)			0.649 (0.038)	*0.056 (0.073)*	
	GAD	0.688 (0.035)			0.591 (0.043)	0.313 (0.064)	
	MDE	0.796 (0.029)			0.643 (0.048)	0.512 (0.072)	
	DYS	0.860 (0.030)			0.674 (0.064)	0.691 (0.083)	
	PTSD	0.634 (0.040)			0.572 (0.043)	0.151 (0.071)	
	AGO		0.833 (0.032)		0.811 (0.036)	−0.240 (0.085)	
	PD		0.738 (0.038)		0.706 (0.038)	*−0.115 (0.078)*	
	SOP		0.742 (0.028)		0.710 (0.030)	*−0.117 (0.066)*	
	SP		0.652 (0.032)		0.643 (0.037)	−0.262 (0.067)	
	ALC			0.689 (0.038)	0.235 (0.035)		0.697 (0.042)
	DRG			0.819 (0.039)	0.291 (0.041)		0.806 (0.044)
	APD			0.774 (0.050)	0.455 (0.049)		0.521 (0.051)

MAN, mania; GAD, generalized anxiety disorder; MDE, major depressive episode; DYS, dysthymia; PSTD, posttraumatic stress disorder; AGO, agoraphobia; PD, panic disorder; SOP, social phobia; SP, specific phobia; ALC, alcohol abuse; DRG, drug abuse; APD, antisocial personality disorder. Nonsignificant loadings (p > 0.05) are in italics.

Because the data were comprised of binary measures indicating the presence or absence of a psychiatric disorder, each disorder had a single measurement occasion-specific threshold that was a direct function of its prevalence rate. The occasion-specific thresholds were, therefore, equal in the bifactor and the correlated factors model. Adding threshold constraints did not significantly worsen the models’ fits (steps 3a and 3b), meaning that threshold invariance was supported for both models. The bifactor model and correlated factors model thus exhibited an equal degree of strong longitudinal invariance. The fully constrained model parameters for both the models are presented in **Table 3**.

**Table 3:** Constrained factor loadings (standard errors in parentheses) and disorder prevalence for the longitudinal correlated factors model and the longitudinal bifactor model.

Disorder	Correlated factors model			Bifactor model			Prevalence^†^
	Distress	Fear	Externalizing	General	Internalizing	Externalizing	
MAN	0.754 (0.025)			0.705 (0.027)	*0.103 (0.063)*		2.8%
GAD	0.719 (0.023)			0.646 (0.030)	0.259 (0.058)		5.7%
MDE	0.821 (0.018)			0.720 (0.036)	0.446 (0.064)		15.6%
DYS	0.785 (0.019)			0.664 (0.047)	0.580 (0.062)		4.8%
PTSD	0.613 (0.027)			0.577 (0.028)	*0.094 (0.054)*		5.1%
AGO		0.783 (0.024)		0.745 (0.031)	−0.286 (0.065)		4.5%
PD		0.759 (0.026)		0.709 (0.026)	*−0.092 (0.065)*		3.3%
SOP		0.724 (0.020)		0.686 (0.025)	−0.216 (0.058)		11.7%
SP		0.702 (0.022)		0.674 (0.027)	−0.276 (0.058)		11.3%
ALC			0.766 (0.024)	0.256 (0.026)		0.762 (0.028)	17.7%
DRG			0.898 (0.027)	0.324 (0.029)		0.843 (0.032)	7.1%
APD			0.751 (0.031)	0.424 (0.036)		0.545 (0.032)	3.2%
Lagged factor correlation (standard error)^‡^	0.573 (0.032)	0.644 (0.036)	0.640 (0.030)	0.649 (0.024)	0.327 (0.072)	0.643 (0.035)	

MAN, mania; GAD, generalized anxiety disorder; MDE, major depressive episode; DYS, dysthymia; PSTD, posttraumatic stress disorder; AGO, agoraphobia; PD, panic disorder; SOP, social phobia; SP, specific phobia; ALC, alcohol abuse; DRG, drug abuse; APD, antisocial personality disorder. Nonsignificant loadings (p > 0.05) are in italics. Lagged indicator correlations (residual correlations) are presented in **Supplementary Table 5***.* *^†^Prevalance estimates are based on estimated model thresholds.* *^‡^Correlation of factor values between time 1 and time 2. For a full presentation of all factor correlations, see* **Supplementary Table 6***.*

The bifactor model parameters presented in **Table 3** were obtained from a model that included only homotypic factor correlations over time. A bifactor model with heterotypic factor correlations was also fitted (for correlations, see Supplementary Material). The inclusion of heterotypic factor correlations had no effect on model fit, and the more parsimonious bifactor model with only homotypic correlations was preferred. Furthermore, examining the correlations of the specific internalizing factor was considered not meaningful because the factor was not well defined. The results of invariance tests were the same regardless of whether the longitudinal bifactor model included heterotypic correlations or not.

### Factor Structure of Change in Psychopathology

Parallel analysis indicated three underlying factors also in the change score data. A correlated three-factor model and a bifactor model with two specific factors were first estimated using EFA and then using CFA (for factor loadings, see **Supplementary Tables 7** and **8**). In CFA, the bifactor model demonstrated acceptable model fit (CFI = 0.959, TLI = 0.935, RMSEA = 0.025), whereas the fit of the correlated factors was poor (CFI = 0.903, TLI = 0.874, RMSEA = 0.035). The general factor of change exhibited acceptable construct reliability (*H* = 0.72), but other latent factors in the bifactor model or in the correlated factors model were not reliably defined in change score data (*H* range 0.46–0.67). A further inspection of correlations among the disorders revealed considerable positive correlations between disorders belonging to different factors of the correlated factors model, showing that the diagnostic statuses changed in the same direction across the fear, distress, and externalizing factors (see **Supplementary Figure 1** for the correlations).

The model fit difference between the bifactor and the correlated factors model was more notable in the change score relative to the cross-sectional data (ΔCFI = 0.056, ΔTLI = 0.061, and ΔRMSEA = 0.010 in the change-score analysis compared to a ΔCFI = 0.011, ΔTLI = 0.011, and ΔRMSEA = 0.005 in the cross-sectional analysis; see **Supplementary Table 9** for the comparison of model fit indices). Further examination of factor congruence coefficients (26) indicated that the specific internalizing factor in the bifactor model had a very low degree of congruence (ϕ = 0.18) between change score and cross-sectional data, suggesting that the pattern of specific internalizing factor loadings was different between the datasets. In comparison, the general factor and the specific externalizing factors were congruent across change score and cross-sectional data (ϕ = 0.98 and ϕ = 0.99, respectively).

**Figure 2:**
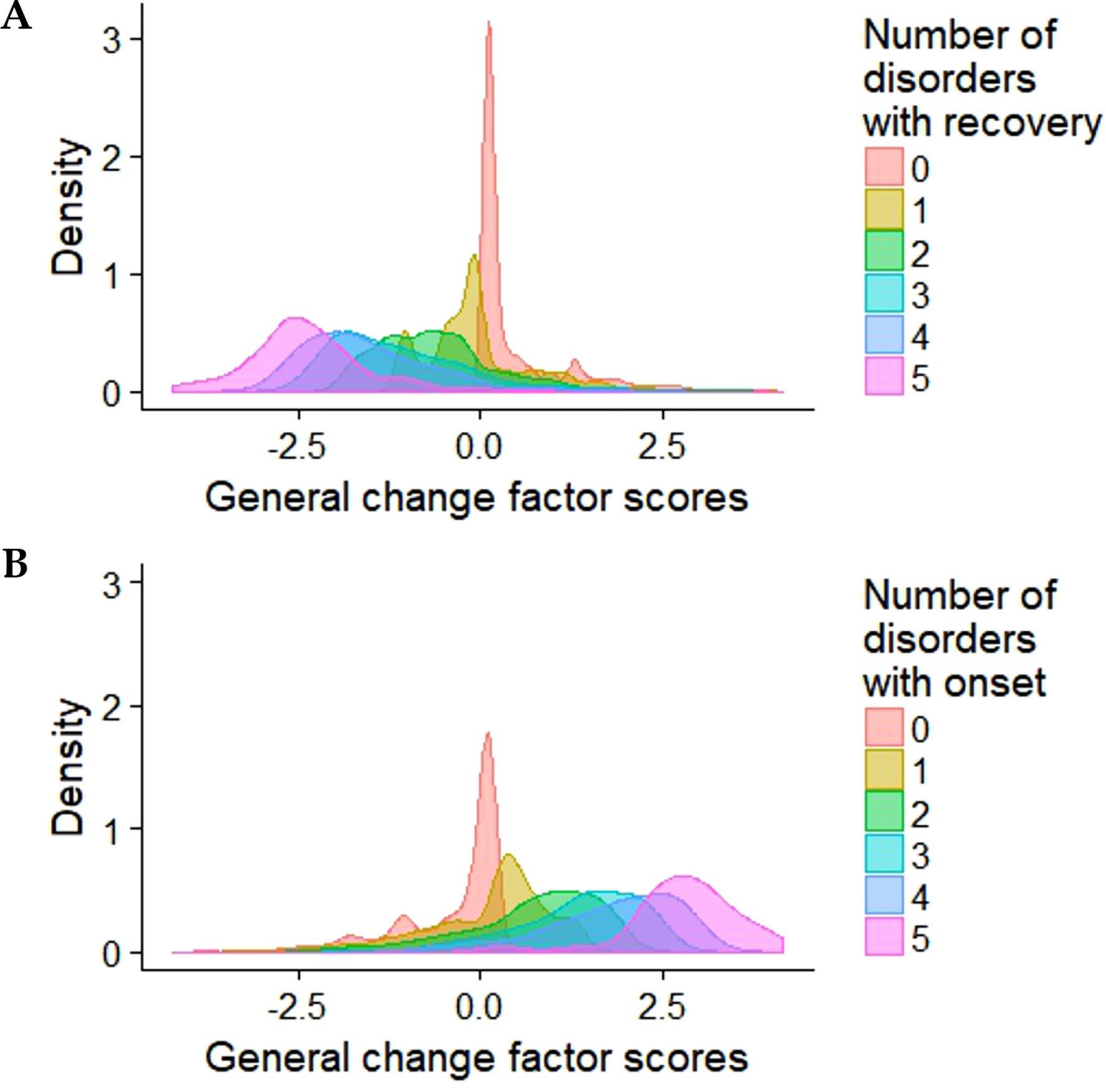
The general factor scores by the frequency of disorder recovery **(A)** and onset **(B)**.

To facilitate interpretation of the factors *via post hoc* analysis, we further investigated the bifactor model of disorder change scores by analyzing the factors’ demographic correlates and their associations with disorder onset, recovery, and recurrence. First, we regressed the factors on respondent age and gender within a single structural equation model. Higher age was associated with decreases in the general factor (β = −0.075, *p* = 0.007) and externalization factor (β = −0.171, *p* < 0.001). Gender was not associated with any of the latent factors for changes. Next, counts of disorder onsets and recoveries between T1 and T2 were calculated for each respondent and categorized into six levels from 0 = *no change in disorder status* to 5 = *onset or recovery from five or more disorders*. A count of disorder recurrence was also calculated and categorized into four levels from 0 = *no recurring disorders* to 3 = *recurrence of three or more disorders*. Analysis of variance indicated significant differences (*p* < 0.001) with large effect sizes in the predicted general change factor scores between different frequencies of disorder onset and recovery (effect size estimate η² = 0.39 for onset and η² = 0.42 for recovery). In a dose–response manner, the general change factor score increased as the number of disorder onsets grew larger and decreased as the number of disorder recoveries grew larger (**Table 4**, **Figure 2**). There were no significant differences in the general change factor scores by increasing levels of disorder recurrence, nor did the specific internalizing and externalizing change factor scores differ considerably across levels of disorder onset and recovery (data available upon request).

**Table 4:** General psychopathology factor extracted from change score data: Mean (M) factor scores and standard deviations (SD) by the number of disorders with recovery, disorders with onset, and disorders with recurrence.

	Number of disorders	M	SD	n	%
Disorders with recovery					
	0	0.31	0.53	2419	48%
	1	−0.09	0.62	1361	27%
	2	−0.54	0.67	692	14%
	3	−0.94	0.74	302	6%
	4	−1.29	0.66	129	3%
	5+	−1.93	0.64	98	2%
					100%
Disorders with onset					
	0	−0.38	0.56	3030	61%
	1	0.03	0.65	1101	22%
	2	0.49	0.71	480	10%
	3	0.97	0.67	217	4%
	4	1.34	0.70	113	2%
	5+	2.09	0.58	60	1%
					100%
Disorders with recurrence					
	0	−0.08	0.72	3743	75%
	1	−0.07	0.89	870	17%
	2	−0.04	1.01	252	5%
	3+	0.03	1.00	136	3%
					100%

## Discussion

This study tested the structural stability (i.e., longitudinal invariance) of a bifactor model of psychiatric comorbidity that includes a general psychopathology factor, as well as examined the models’ generalizability to change processes in psychopathology. The findings were compared to a correlated factors model that explains psychiatric comorbidity with correlated underlying factors but has no general psychopathology factor. Both the bifactor model and the correlated factors model displayed strong structural stability over time, meaning that the relationships between psychiatric disorders and the modeled latent factors remained stable over the 10-year period in adulthood. However, the bifactor model explained the observed structure of temporal changes in disorder status better than the correlated factors model. To our knowledge, these findings show for the first time that the general psychopathology factor’s meaning stays rather immutable over time and that it can be used to model development of and recovery from psychopathology in adulthood.

We found that individual differences in intraindividual patterns of change in psychopathology were characterized by a rather similar bifactor structure as the cross-sectional individual differences. Furthermore, the observed structure of temporal changes in disorder status supported the bifactor model over the traditional correlated factors model, suggesting that change in psychopathology results from one general liability factor to which specific, or “pure,” internalizing and externalizing tendencies give color, rather than from correlated primary internalizing and externalizing tendencies. The strength of the bifactor model thus appears to lie in its ability to separate general psychopathology from the specific residual factors, making it better-suited than the correlated factors model to describe the structure of changes in psychiatric disorders.

The present findings contribute to the recent discussion on the utility of the general psychopathology factor. The findings that the bifactor structure is stable and fits change data relatively better than the correlated factors model speak against the notion of general psychopathology factor being only a statistical artifact (9, 10). The results are also in line with previous evidence supporting the validity and utility of the general factor (27). The general factor robustly correlates with external variables such as demographic factors, negative emotionality, and cognitive ability (1, 28), and higher scores on the factor predict future psychopathology and life impairment (2, 3). Prior work has also demonstrated that the best-fitting structural model for genetic risk factors for psychopathology includes a general genetic factor (6). The general psychopathology factor, therefore, reflects the influence of genetic risk factors that are shared by multiple disorders, although this does not automatically implicate a pure genetic etiology (7, 29, 30). Our finding that the bifactor structure describes changes in disorder status suggest that there is important within-individual variation in the general psychopathology risk as well.

Once the general psychopathology factor was included in the factor structure, the specific internalizing factor was not reliably specified, and some of its the loadings became nonsignificant and/or negative. For example, in our cross-sectional examination of the bifactor model, the specific internalizing factor represented either high distress or high fear, depending on the measurement occasion. Correspondingly, we also observed diminishing general factor loadings among disorders reflecting distress and increasing loadings among disorders reflecting fear over time—a pattern similar to that reported previously (13). Prior work has documented similar anomalous findings also with regard to the subfactors of specific externalizing (28). The results are not that surprising given that the specific factors are residualized factors, and such anomalous results, in which the loadings on a specific factor differ strongly, are generally not uncommon when applying bifactor models (31). As was evident, longitudinal models hold the potential to resolve some of the technical weaknesses of bifactor models, as the loading indeterminacy of cross-sectional analyses got resolved in longitudinal modeling that implies drastically higher degrees of freedom. When examined longitudinally, the specific internalizing factor loadings were rather similar across both measurements and represented high distress and low fears.

To our knowledge, this is the first study to investigate the longitudinal invariance of the bifactor model of comorbidity and to evaluate the generalizability of structural comorbidity models to change processes in psychopathology. Several limitations should be considered when interpreting the results. First, although in terms of model fit indices, our results favored the bifactor model over the traditional correlated factors model, the question remains open as to when such differences become practically significant. Therefore, even though the findings support the bifactor conceptualization, they do not disprove the potential relevance of the correlated factors model. Second, the measurement of change in psychopathology was crude in that it did not reflect exact times of onset or recovery. Nevertheless, our change scores provided a simple-to-understand approach for examining change processes in psychopathology and were modeled as continuous-valued underlying changes using the liability-threshold model, which is the standard approach in structural equation modeling of ordinal-valued data. Third, we used DSM-III-R and DSM-IV rather than DSM-5 diagnoses, which means that the generalizability of the results to the most recent diagnostic criteria might be limited. Fourth, while the sample was representative of the general US population, the results may not generalize to populations outside the USA. Fifth, due to limited data on psychotic disorders, we could not extend the structural models to include a thought disorder factor that has been found in some other datasets (32, 33). Future studies covering a wider range of psychiatric disorders, and perhaps also pathological personality traits (7), are warranted to confirm and extend our findings.

In conclusion, these findings highlight the utility of the bifactor model in understanding the structure of psychiatric comorbidity. The bifactor structure of psychopathology is stable over time, and both the cross-sectional pattern and the pattern of change in psychiatric comorbidity follow a bifactor structure. The finding that the structure with a general psychopathology factor describes both cross-sectional differences between individuals and differences in how individuals change suggests that the general psychopathology factor may represent a transdiagnostic entity that can both trigger and dissolve a range of psychiatric disorders. The general psychopathology factor implies that shared psychopathological mechanisms operate across multiple mental disorders. By identifying these shared mechanisms, it may be possible to develop interventions that focus on the common processes rather than diagnosis-specific symptoms. Emerging evidence suggests that such transdiagnostic treatment protocols may be as efficient as diagnosis-specific treatments (34). Future research on therapeutic change could benefit from using the bifactor model to disentangle the effects of both general and specific therapeutic processes (35).

## Data Availability

The dataset analyzed for this study is publicly available at the Inter-university Consortium for Political and Social Research website (www.icpsr.umich.edu).

## Author Contributions

TR, MJ, and KG developed the study concept. KG performed the data analysis and drafted the paper, and TR and MJ provided critical revisions. All authors approved the final version of the paper for submission.

## Conflict of Interest Statement

The authors declare that the research was conducted in the absence of any commercial or financial relationships that could be construed as a potential conflict of interest.
