# Supplementary material for: The General Psychopathology Factor: Structural Stability and Generalizability to Within-Individual Changes
Source: Front Psychiatry. 2019 Aug 30;10:594. doi: 10.3389/fpsyt.2019.00594 (PMC6728891; doi:10.3389/fpsyt.2019.00594)
Supplement: Supplementary file 1 [file DataSheet_1.docx]

Supplementary Material

# Cross-sectional factor structure

**Supplementary Table 1.** Standardized factor loadings from exploratory factor analysis of the cross-sectional structure of psychopathology using Geomin and Bi-geomin rotation criterions

|  |  | Geomin rotation | | | Bi-geomin rotation | | |
| --- | --- | --- | --- | --- | --- | --- | --- |
| Time | Diagnosis | Factor 1  “Distress” | Factor 2  “Fear” | Factor 3  “Externalizing” | Factor 1  “General” | Factor  “Internalizing” | Factor 3  “Externalizing” |
| T1 | MAN | **0.500** | 0.237 | 0.194 | **0.745** | -0.005 | 0.160 |
|  | GAD | **0.680** | 0.072 | 0.028 | **0.723** | -0.150 | 0.012 |
|  | MDE | **0.845** | -0.005 | 0.027 | **0.817** | -0.239 | 0.013 |
|  | DYS | **0.960** | -0.237 | -0.009 | **0.724** | **-0.402** | -0.009 |
|  | PTSD | **0.356** | 0.222 | 0.076 | **0.552** | 0.026 | 0.053 |
|  | AGO | 0.068 | **0.731** | -0.020 | **0.659** | **0.391** | -0.062 |
|  | PD | **0.385** | **0.445** | -0.042 | **0.721** | 0.142 | -0.071 |
|  | SOP | -0.002 | **0.671** | 0.151 | **0.603** | **0.379** | 0.102 |
|  | SP | -0.004 | **0.772** | 0.046 | **0.647** | **0.435** | -0.001 |
|  | ALC | -0.080 | 0.011 | **0.882** | 0.248 | 0.036 | **0.825** |
|  | DRG | 0.020 | -0.028 | **0.890** | **0.315** | -0.013 | **0.833** |
|  | APD | 0.156 | -0.001 | **0.616** | **0.369** | -0.039 | **0.573** |
|  |  |  |  |  |  |  |  |
| T2 | MAN | 0.272 | **0.355** | 0.210 | **0.641** | 0.021 | 0.205 |
|  | GAD | **0.551** | 0.196 | -0.025 | **0.629** | 0.224 | -0.012 |
|  | MDE | **0.926** | 0.008 | -0.109 | **0.735** | **0.500** | -0.087 |
|  | DYS | **0.942** | -0.090 | 0.016 | **0.710** | **0.557** | 0.025 |
|  | PTSD | **0.377** | 0.194 | 0.077 | **0.525** | 0.135 | 0.079 |
|  | AGO | -0.014 | **0.931** | -0.064 | **0.814** | **-0.384** | -0.040 |
|  | PD | 0.136 | **0.640** | -0.007 | **0.696** | -0.181 | 0.008 |
|  | SOP | 0.128 | **0.574** | 0.113 | **0.679** | -0.152 | 0.116 |
|  | SP | -0.025 | **0.669** | 0.027 | **0.603** | -0.280 | 0.037 |
|  | ALC | -0.003 | -0.115 | **0.771** | 0.213 | 0.094 | **0.699** |
|  | DRG | -0.140 | 0.005 | **0.928** | 0.273 | -0.020 | **0.843** |
|  | APD | 0.026 | 0.158 | **0.560** | **0.400** | -0.013 | **0.513** |

*Note*. MAN=mania, GAD=generalized anxiety disorder, MDE=major depressive episode, DYS=dysthymia, PSTD=post-traumatic stress disorder, AGO=agoraphobia, PD=panic disorder, SOP=social phobia, SP=specific phobia, ALC=alcohol abuse, DRG=drug abuse, APD=anti-social personality disorder. Loadings of | ≥ 0.315| are bolded. In a correlated factors exploratory model, the pattern of loadings revealed internalizing fear and distress factors and an externalizing factor. A bifactor rotation criterion (1) identified a general factor and internalizing- and externalizing-specific factors with no cross-loadings between the internalizing and externalizing. As the specific internalizing factor included both positive and negative loadings and some of the loadings were low in magnitude, a four-factor model with a bifactor rotation criterion was also fitted (results available upon request). Adding a third specific factor did not improve the interpretability of the bifactor solution.

**Supplementary Table 2.** Model fit indices for alternative CFA models of the cross-sectional structure of psychopathology

| Time | Fit indice | One-factor  model | Correlated  two-factor  model | Correlated  three-factor  model | Bifactor  model (general factor and two specific factors) |
| --- | --- | --- | --- | --- | --- |
| T1 | Chi-square (df) | 1267.605 (54) | 294.436 (53) | 179.757 (51) | 104.781 (42) |
|  | CFI | 0.785 | 0.957 | 0.978 | 0.989 |
|  | TLI | 0.737 | 0.947 | 0.972 | 0.983 |
|  | RMSEA | 0.067 | 0.030 | 0.022 | 0.017 |
|  |  |  |  |  |  |
|  | AIC^†^ |  |  | 27520.196 | 27452.746 |
|  | BIC^†^ |  |  | 27696.166 | 27687.372 |
|  |  |  |  |  |  |
|  |  |  |  |  |  |
| T2 | Chi-square (df) | 697.343 (54) | 326.592 (53) | 189.960 (51) | 84.729 (42) |
|  | CFI | 0.845 | 0.934 | 0.967 | 0.990 |
|  | TLI | 0.811 | 0.918 | 0.957 | 0.984 |
|  | RMSEA | 0.049 | 0.032 | 0.023 | 0.014 |
|  |  |  |  |  |  |
|  | AIC^†^ |  |  | 26960.401 | 26856.441 |
|  | BIC^†^ |  |  | 27136.371 | 27091.067 |

Note. ^†^AIC and BIC values were obtained using robust maximum likelihood (MLR) estimation. Model fit indices for a bifactor model with a general factor and three specific factors are not presented because the solution was inadmissible.

**Supplementary Table 3.** Standardized factor loadings (standard errors in parentheses) of the correlated factors model and the bifactor model in cross-sectional CFA

| Time | Disorder | Correlated factors model | | |  | Bifactor model | | |
| --- | --- | --- | --- | --- | --- | --- | --- | --- |
|  |  | Distress | Fear | Externalizing |  | General | Internalizing | Externalizing |
| T1 | MAN | 0.792 (0.032) |  |  |  | 0.763 (0.034) | *0.090 (0.112)* |  |
|  | GAD | 0.745 (0.029) |  |  |  | 0.736 (0.029) | *-0.016 (0.095)* |  |
|  | MDE | 0.841 (0.022) |  |  |  | 0.845 (0.026) | *-0.100 (0.105)* |  |
|  | DYS | 0.739 (0.026) |  |  |  | 0.777 (0.039) | -0.264 (0.100) |  |
|  | PTSD | 0.577 (0.036) |  |  |  | 0.547 (0.038) | *0.113 (0.077)* |  |
|  | AGO |  | 0.740 (0.032) |  |  | 0.564 (0.065) | 0.516 (0.079) |  |
|  | PD |  | 0.783 (0.033) |  |  | 0.666 (0.045) | 0.289 (0.091) |  |
|  | SOP |  | 0.707 (0.026) |  |  | 0.548 (0.055) | 0.455 (0.072) |  |
|  | SP |  | 0.739 (0.027) |  |  | 0.559 (0.064) | 0.540 (0.077) |  |
|  | ALC |  |  | 0.809 (0.028) |  | 0.280 (0.031) |  | 0.798 (0.035) |
|  | DRG |  |  | 0.922 (0.029) |  | 0.344 (0.034) |  | 0.838 (0.037) |
|  | APD |  |  | 0.716 (0.035) |  | 0.397 (0.045) |  | 0.552 (0.043) |
|  |  |  |  |  |  |  |  |  |
| T2 | MAN | 0.690 (0.035) |  |  |  | 0.660 (0.040) | *0.130 (0.095)* |  |
|  | GAD | 0.685 (0.032) |  |  |  | 0.553 (0.059) | 0.374 (0.083) |  |
|  | MDE | 0.828 (0.027) |  |  |  | 0.576 (0.089) | 0.668 (0.093) |  |
|  | DYS | 0.860 (0.028) |  |  |  | 0.565 (0.093) | 0.708 (0.089) |  |
|  | PTSD | 0.571 (0.038) |  |  |  | 0.491 (0.053) | 0.244 (0.081) |  |
|  | AGO |  | 0.837 (0.032) |  |  | 0.846 (0.037) | *-0.141 (0.121)* |  |
|  | PD |  | 0.743 (0.037) |  |  | 0.715 (0.037) | *-0.008 (0.105)* |  |
|  | SOP |  | 0.747 (0.027) |  |  | 0.718 (0.028) | *-0.011 (0.097)* |  |
|  | SP |  | 0.638 (0.032) |  |  | 0.652 (0.035) | *-0.134 (0.095)* |  |
|  | ALC |  |  | 0.702 (0.041) |  | 0.255 (0.036) |  | 0.691 (0.057) |
|  | DRG |  |  | 0.835 (0.042) |  | 0.329 (0.045) |  | 0.810 (0.065) |
|  | APD |  |  | 0.733 (0.050) |  | 0.439 (0.052) |  | 0.480 (0.054) |

*Note.* MAN=mania, GAD=generalized anxiety disorder, MDE=major depressive episode, DYS=dysthymia, PSTD=post-traumatic stress disorder, AGO=agoraphobia, PD=panic disorder, SOP=social phobia, SP=specific phobia, ALC=alcohol abuse, DRG=drug abuse, APD=anti-social personality disorder. Nonsignificant loadings (*p*>.05) are in italics. Once the general factor was modeled, the specific internalizing factor captured variance specific to either fear (T1) or distress (T2). Correspondingly, the general factor loadings of disorders reflecting distress became slightly weaker over time, whereas the loadings of disorders reflecting fear grew stronger. With increasing age, the general factor thus reflected disorders in the fear domain to some degree more strongly than disorders in the distress domain.

**Supplementary Table 4.** Construct reliability (*H*) for the correlated factors model and the bifactor model in cross-sectional data (T1/T2)

|  | Correlated factors model | | | Bifactor model | | |
| --- | --- | --- | --- | --- | --- | --- |
|  | Distress | Fear | Externalizing | General | Internalizing | Externalizing |
| *H* coefficient | 0.88/0.88 | 0.83/0.85 | 0.90/0.82 | 0.91/0.89 | 0.55/0.68 | 0.82/0.76 |

*Note.* Construct reliability (*H*) coefficient reflects the how well a latent variable is represented by the indicators and how replicable the factor is across studies. High *H* values (>.70) indicate a well-defined latent factor (see Hancock & Mueller, 2001).

**2 Longitudinal invariance**

**Supplementary Table 5.** Indicator residual correlations in the fully constrained correlated factors model and bifactor model

| Disorder | Correlated factors model | Bifactor model |
| --- | --- | --- |
| MAN | *0.033* | *0.034* |
| GAD | *0.049* | *0.052* |
| MDE | *0.012* | *-0.003* |
| DYS | *-0.001* | *-0.044* |
| PTSD | 0.147 | 0.143 |
| AGO | *0.052* | *0.060* |
| PD | *0.014* | *0.056* |
| SOP | 0.093 | 0.110 |
| SP | 0.215 | 0.212 |
| ALC | 0.119 | 0.078 |
| DRG | *0.057* | *0.048* |
| APD | 0.291 | 0.344 |

*Note.* MAN=mania, GAD=generalized anxiety disorder, MDE=major depressive episode, DYS=dysthymia, PSTD=post-traumatic stress disorder, AGO=agoraphobia, PD=panic disorder, SOP=social phobia, SP=specific phobia, ALC=alcohol abuse, DRG=drug abuse, APD=anti-social personality disorder, Nonsignificant correlations (*p*>.05) are in italics.

**Supplementary Table 6.** Factor correlations (standard errors) in the fully constrained correlated factors model and bifactor model

|  | Correlated factors model | | | | |
| --- | --- | --- | --- | --- | --- |
|  | Fear T1 | Externalizing T1 | Distress T2 | Fear T2 | Externalizing T2 |
| Distress T1 | 0.741 (0.029) | 0.414 (0.033) | 0.573 (0.032) | 0.552 (0.034) | 0.192 (0.041) |
| Fear T1 |  | 0.296 (0.036) | 0.418 (0.035) | 0.644 (0.036) | 0.225 (0.038) |
| Externalizing T1 |  |  | 0.240 (0.037) | 0.265 (0.037) | 0.640 (0.030) |
| Distress T2 |  |  |  | 0.687 (0.032) | 0.323 (0.035) |
| Fear T2 |  |  |  |  | 0.365 (0.044) |
|  |  |  |  |  |  |
|  | Bifactor model | | | | |
|  | General T2 | Internalizing T2 | Externalizing T2 |  | |
| General T1 | 0.641 (0.027) | -0.149 (0.052) | *-0.021 (0.033)* | |  |
| Internalizing T1 | *0.011 (0.046)* | 0.369 (0.073) | *-0.030 (0.049)* | |  |
| Externalizing T1 | *0.031 (0.030)* | *-0.027 (0.054)* | 0.641 (0.027) | |  |

*Note*. Nonsignificant correlations (*p*>.05) are in italics.

# Factor structure of change score data

**Supplementary Table 7.** Standardized factor loadings from exploratory factor analysis of the structure of change in psychopathology using Geomin and Bi-geomin rotation criterions

|  | Geomin rotation | | |  | Bi-geomin rotation | | |
| --- | --- | --- | --- | --- | --- | --- | --- |
| Diagnosis | Factor  1 | Factor  2 | Factor  3 |  | Factor  1 | Factor  2 | Factor  3 |
| MAN | 0.140 | **0.379** | 0.013 |  | **0.364** | 0.266 | 0.018 |
| GAD | **0.419** | 0.154 | -0.011 |  | **0.493** | 0.012 | -0.015 |
| MDE | **0.674** | 0.012 | 0.040 |  | **0.666** | -0.169 | 0.026 |
| DYS | **0.740** | -0.140 | -0.005 |  | **0.629** | -0.308 | -0.022 |
| PTSD | 0.152 | 0.035 | 0.098 |  | 0.191 | -0.012 | 0.092 |
| AGO | -0.006 | **0.617** | -0.051 |  | **0.350** | **0.495** | -0.036 |
| PD | 0.154 | **0.398** | 0.011 |  | **0.389** | 0.277 | 0.016 |
| SOP | 0.072 | **0.449** | -0.002 |  | **0.337** | **0.340** | 0.006 |
| SP | -0.047 | **0.473** | 0.003 |  | 0.238 | **0.391** | 0.014 |
| ALC | -0.011 | 0.002 | **0.616** |  | 0.142 | 0.007 | **0.597** |
| DRG | 0.003 | -0.004 | **0.681** |  | 0.167 | -0.002 | **0.660** |
| APD | 0.075 | 0.000 | **0.318** |  | 0.151 | -0.019 | 0.307 |

*Note.* MAN=mania, GAD=generalized anxiety disorder, MDE=major depressive episode, DYS=dysthymia, PSTD=post-traumatic stress disorder, AGO=agoraphobia, PD=panic disorder, SOP=social phobia, SP=specific phobia, ALC=alcohol abuse, DRG=drug abuse, APD=anti-social personality disorder. Loadings of | ≥ 0.315| are bolded. For APD, change scores reflected change in lifetime diagnosis.

**Supplementary Table 8.** Standardized factor loadings (standard errors) of the correlated factors model and the bifactor model in CFA with change score data

| Disorder | Correlated factors model | | |  | Bifactor model | | |
| --- | --- | --- | --- | --- | --- | --- | --- |
|  | Distress | Fear | Externalizing |  | General | Internalizing | Externalizing |
| MAN | 0.412 (0.028) |  |  |  | 0.388 (0.044) | 0.233 (0.074) |  |
| GAD | 0.514 (0.024) |  |  |  | 0.490 (0.022) | *-0.030 (0.079)* |  |
| MDE | 0.696 (0.024) |  |  |  | 0.659 (0.043) | -0.232 (0.107) |  |
| DYS | 0.570 (0.024) |  |  |  | 0.590 (0.059) | -0.349 (0.095) |  |
| PTSD | 0.206 (0.028) |  |  |  | 0.199 (0.027) | *-0.027 (0.042)* |  |
| AGO |  | 0.574 (0.024) |  |  | 0.385 (0.071) | 0.463 (0.066) |  |
| PD |  | 0.516 (0.029) |  |  | 0.412 (0.044) | 0.245 (0.067) |  |
| SOP |  | 0.491 (0.023) |  |  | 0.364 (0.050) | 0.313 (0.062) |  |
| SP |  | 0.409 (0.025) |  |  | 0.271 (0.059) | 0.370 (0.049) |  |
| ALC |  |  | 0.606 (0.040) |  | 0.154 (0.026) |  | 0.583 (0.055) |
| DRG |  |  | 0.682 (0.044) |  | 0.176 (0.027) |  | 0.675 (0.062) |
| APD |  |  | 0.347 (0.030) |  | 0.154 (0.036) |  | 0.295 (0.032) |

*Note*. MAN=mania, GAD=generalized anxiety disorder, MDE=major depressive episode, DYS=dysthymia, PSTD=post-traumatic stress disorder, AGO=agoraphobia, PD=panic disorder, SOP=social phobia, SP=specific phobia, ALC=alcohol abuse, DRG=drug abuse, APD=anti-social personality disorder. Nonsignificant loadings (*p*>.05) are in italics. For APD, change scores reflected change in lifetime diagnosis.

**Supplementary Table 9.** Differences in model fit indices for the correlated factors model and the bifactor model

in cross-sectional and change score data

|  | Cross-sectional data (T1) | | |  | Change score data | | |
| --- | --- | --- | --- | --- | --- | --- | --- |
|  | Correlated factors model | Bifactor model | Difference  (Bifactor model – correlated factors model) |  | Correlated factors model | Bifactor model | Difference  (Bifactor model – correlated factors model) |
| Chi-square (df) | 179.757 (51) | 104.781 (42) |  |  | 358.007 (51) | 171.686(42) |  |
| CFI | 0.978 | 0.989 | 0.011 |  | 0.903 | 0.959 | 0.056 |
| TLI | 0.972 | 0.983 | 0.011 |  | 0.874 | 0.935 | 0.061 |
| RMSEA | 0.022 | 0.017 | -0.005 |  | 0.035 | 0.025 | -0.010 |
|  |  |  |  |  |  |  |  |
| AIC^†^ | 27520.196 | 27452.746 | -67.45 |  | 46555.476 | 46469.374 | -86.102 |
| BIC^†^ | 27696.166 | 27687.372 | -8.794 |  | 46809.655 | 46782.209 | -27.446 |

*Note.* ^†^ AIC and BIC values were obtained using robust maximum likelihood (MLR) estimation. High values of CFI and TLI and low values of RMSEA indicate good fit. Lower values of AIC and BIC indicate better model fit. The larger the difference in AIC and BIC between the models, the stronger the evidence in favor of the model with the lower AIC or BIC. Conventional guidelines suggest that a BIC difference of 6 to 10 constitutes a strong evidence, and a difference of 10 or more constitutes a very strong evidence for the superiority of one model over the other (3).


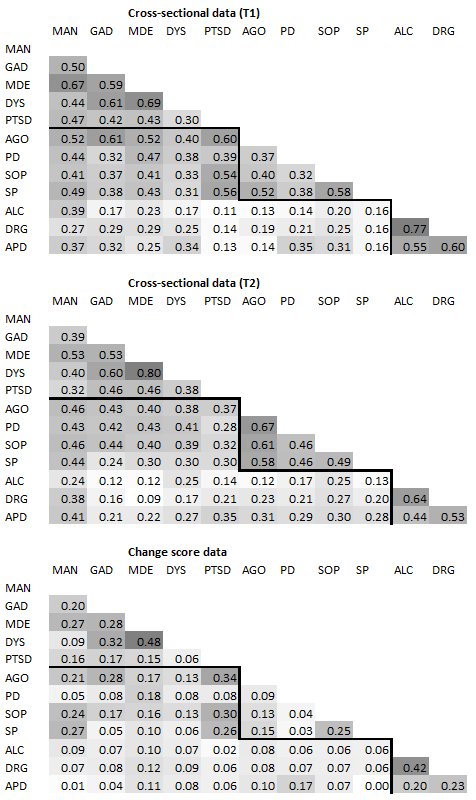


**Supplementary Figure 1.** Tetrachoric correlations between disorder statuses in cross-sectional and change score data. MAN=mania, GAD=generalized anxiety disorder, MDE=major depressive episode, DYS=dysthymia, PSTD=post-traumatic stress disorder, AGO=agoraphobia, PD=panic disorder, SOP=social phobia, SP=specific phobia, ALC=alcohol abuse, DRG=drug abuse, APD=anti-social personality disorder.

**Supplementary references**

1. Jennrich RI, Bentler PM. Exploratory Bi-Factor Analysis. Psychometrika. 2011 Oct 9;76(4):537–49.

2. Hancock GR, Mueller RO. Rethinking construct reliability within latent variable systems. In: Cudeck R, du Toit S, Sorbom D, editors. Structural equation modeling: Present and future. Lincolnwood, IL: Scientific Software International; 2001. p. 195–216.

3. Raftery AE. Bayesian Model Selection in Social Research. Sociol Methodol. 1995;25:111.
